# Supplementary material for: Variation in purple sea urchin (Strongylocentrotus purpuratus) morphological traits in relation to resource availability
Source: PeerJ. 2021 Apr 27;9:e11352. doi: 10.7717/peerj.11352 (PMC8086578; doi:10.7717/peerj.11352)
Supplement: Supplemental Information 4 — These data were generated from benthic subtidal surveys led by Joshua G. Smith. [file peerj-09-11352-s004.rtf]

This file explains all of the variables in each of the datasets required to conduct the analyses used in the manuscript, “Variation in purple sea urchin (Strongylocentrotus purpuratus) morphological traits in relation to resource availability.” These data were generated from benthic subtidal surveys led by Joshua G. Smith.————————————————————————————————————******Information about data file “Urchin_data.csv”******This data file contains information on habitat attributes and sea urchin morphometrics collected from benthic surveys along the Monterey Peninsula, CA, USA in the year 2017. All data reported are summarized at the size level, reported as mean values recorded from 1x1m quadrats. COLUMN HEADINGSYear = survey yearSite Number = site nameDensity = mean density of purple sea urchins summarized across 16 1x1m quadratsBrown = proportion cover brown algae summarized across 13 1x1m quadrats and 16 UPC points per quadratEncrusting = proportion cover encrusting algae summarized across 13 1x1m quadrats and 16 UPC points per quadratRed = proportion cover red algae summarized across 13 1x1m quadrats and 16 UPC points per quadratTH = mean urchin test height (mm)TD = mean urchin test diameter (mm)LL = mean urchin lantern length (mm)LW = mean urchin lantern width (mm)LI = mean urchin lantern index (LL / TD)GI = mean urchin gonad index ((gonad mass (g) / animal mass (g))*100)ZONE = categorical assignment for forest (FOR) or barren (BAR)GWM = mean gonad wet mass (g)Log_GWM = log-transformed GWMLog_TD = log-transformed TD————————————————————————————————————******Information about the data file “zone_data.csv”******This data file contains data summarized by test Log_TD for plotting the relationship between lantern length and gonad weight. See description above for column headings. ————————————————————————————————————******Information about the data file “Smith_raw_data_file.csv”******This data file contains unsummarized, raw data. Each row is a single individual purple sea urchin. COLUMN HEADINGSCollection Date = date the urchin was collected from the fieldDate Processed = date the urchin was dissectedYear = survey yearSite Number = site nameTH = test height (mm) TD = test diameter (mm)Log_TD = log-transformed TDUWM = urchin wet mass (g)GWM = gonad wet mass (g)Log_GWM = log-transformed GWMGI = gonad index calculated as (GWM/UWM)*100LL = lantern length (mm)Log_LL = log-transformed LLLW = lantern width (mm). Missing values were not recordedZONE = categorical assignment for forest (FOR) or barren (BAR)————————————————————————————————————******Information about the code file “Smith & Garcia_code.Rmd”******This file contains the code used to analyze the data in , “Variation in purple sea urchin (Strongylocentrotus purpuratus) morphological traits in relation to resource availability.”
